# Supplementary material for: An open-source framework for large-scale, flexible evaluation of biomedical text mining systems
Source: J Biomed Discov Collab. 2008 Jan 29;3:1. doi: 10.1186/1747-5333-3-1 (PMC2276192; doi:10.1186/1747-5333-3-1)
Supplement: Additional file 1 — Evaluation platform source code. The additional file contains the software evaluation framework discussed in this paper. The most current release can be downloaded from SourceForge.net [1]. See the accompanying README file for instructions on installation and use. Also included in the distribution is a collection of UIMA wrappers for some commonly used BioNLP tools and annotated corpora. [file 1747-5333-3-1-S1.zip › bionlp_uima_repository-1.0.1/docs/api/constant-values.html]

Constant Field Values (Center for Computational Pharmacology Util/UIMA API)


|  |  |  |
| --- | --- | --- |
| **Overview** \| Package \| Class \| **Tree** \| **Deprecated** \| **Index** \| **Help** | |  |
| PREV   NEXT | **FRAMES**    **NO FRAMES**     **All Classes** |  |


---


# Constant Field Values


---

**Contents**

- edu.uchsc.\*

edu.uchsc.\*

| edu.uchsc.ccp.uima.ae.nlp.ei.gene.AbGene\_AE | | |
| --- | --- | --- |
| `public static final String` | `PARAM_ABGENE_BASE_DIRECTORY` | `"AbGeneBaseDirectory"` |

| edu.uchsc.ccp.uima.ae.nlp.ei.gene.ABNER\_AE | | |
| --- | --- | --- |
| `public static final String` | `PARAM_USE_BIOCREATIVE_MODEL` | `"UseBioCreativeModel"` |

| edu.uchsc.ccp.uima.ae.nlp.ei.gene.KeX\_AE | | |
| --- | --- | --- |
| `public static final String` | `PARAM_ABGENE_BASE_DIRECTORY` | `"KeXBaseDirectory"` |

| edu.uchsc.ccp.uima.ae.nlp.ei.gene.LingPipeEntityTagger\_AE | | |
| --- | --- | --- |
| `public static final String` | `PARAM_LINGPIPE_ENTITY_MODEL_FILE` | `"entityModelFile"` |
| `public static final String` | `PARAM_LINGPIPE_ENTITY_TYPE` | `"entityType"` |

| edu.uchsc.ccp.uima.ae.nlp.ei.gene.PennBioTagger\_AE | | |
| --- | --- | --- |
| `public static final String` | `PARAM_TAGGER_MODEL_FILE` | `"pennBioTaggerModelFile"` |
| `public static final String` | `PARAM_TAGGER_TYPE` | `"pennBioTaggerType"` |
| `public static final String` | `PARAM_TOKENIZER_MODEL_FILE` | `"pennBioTokenizerModelFile"` |

| edu.uchsc.ccp.uima.ae.nlp.ei.mutation.MutationFinder\_AE | | |
| --- | --- | --- |
| `public static final String` | `PARAM_REGEX_FILE` | `"RegExFile"` |

| edu.uchsc.ccp.uima.ae.nlp.parser.StanfordParser\_AE | | |
| --- | --- | --- |
| `public static final String` | `PARAM_ANNOTATIONSET_VALIDATOR_FILE` | `"AnnotationSetValidatorFile"` |
| `public static final String` | `PARAM_ANNOTATOR_VALIDATOR_FILE` | `"AnnotatorValidatorFile"` |
| `public static final String` | `PARAM_ATTACH_TYPED_DEPENDENCIES` | `"AttachTypedDependencies"` |
| `public static final String` | `PARAM_SERIALIZED_GRAMMAR_FILE` | `"SerializedGrammarFile"` |

| edu.uchsc.ccp.uima.ae.nlp.sentenceDetector.KeXSentenceDetector\_AE | | |
| --- | --- | --- |
| `public static final String` | `PARAM_KEXBASEDIRECTORY` | `"KeXBaseDirectory"` |

| edu.uchsc.ccp.uima.ae.nlp.sentenceDetector.OpenNLPSentenceDetector\_AE | | |
| --- | --- | --- |
| `public static final String` | `PARAM_MODEL_FILE` | `"OpenNlpModelFile"` |

| edu.uchsc.ccp.uima.ae.nlp.tokenizer.GeniaTaggerOutputFileLoader\_AE | | |
| --- | --- | --- |
| `public final String` | `PARAM_GENIA_TAGGER_OUTPUT_FILE` | `"GeniaTaggerOutputFile"` |

| edu.uchsc.ccp.uima.ae.nlp.tokenizer.LingPipePOSTagger\_AE | | |
| --- | --- | --- |
| `public static final String` | `PARAM_LINGPIPE_MODEL_FILE` | `"LingPipeModelFile"` |

| edu.uchsc.ccp.uima.ae.nlp.tokenizer.PennBioTokenizer\_AE | | |
| --- | --- | --- |
| `public static final String` | `PARAM_TOKENIZER_MODEL_FILE` | `"pennTokenizerModelFile"` |

| edu.uchsc.ccp.uima.ae.util.AnnotationFromFileLoader\_AE | | |
| --- | --- | --- |
| `public final String` | `PARAM_ANNOTATION_FILE` | `"AnnotationFile"` |

| edu.uchsc.ccp.uima.ae.util.converter.AnnotationSetOverrider\_AE | | |
| --- | --- | --- |
| `public final String` | `PARAM_ANNOTATION_SETS_TO_IGNORE` | `"AnnotationSetsToIgnore"` |
| `public static final String` | `PARAM_SETDESCRIPTION` | `"SetDescription"` |
| `public static final String` | `PARAM_SETID` | `"SetID"` |
| `public static final String` | `PARAM_SETNAME` | `"SetName"` |

| edu.uchsc.ccp.uima.ae.util.converter.AnnotatorConverter\_AE | | |
| --- | --- | --- |
| `public static final String` | `PARAM_AFFILIATION` | `"Affiliation"` |
| `public static final String` | `PARAM_ANNOTATOR_ID` | `"AnnotatorID"` |
| `public static final String` | `PARAM_ANNOTATOR_IDS_TO_CONVERT` | `"AnnotatorIDsToConvert"` |
| `public static final String` | `PARAM_FIRST_NAME` | `"FirstName"` |
| `public static final String` | `PARAM_LAST_NAME` | `"LastName"` |

| edu.uchsc.ccp.uima.ae.util.converter.AnnotatorOverrider\_AE | | |
| --- | --- | --- |
| `public static final String` | `PARAM_AFFILIATION` | `"Affiliation"` |
| `public static final String` | `PARAM_ANNOTATOR_ID` | `"AnnotatorID"` |
| `public static final String` | `PARAM_FIRST_NAME` | `"FirstName"` |
| `public static final String` | `PARAM_LAST_NAME` | `"LastName"` |

| edu.uchsc.ccp.uima.ae.util.converter.ClassMentionConverter\_AE | | |
| --- | --- | --- |
| `public static final String` | `PARAM_MENTION_TYPES_TO_CONVERT` | `"MentionTypesToConvert"` |
| `public static final String` | `PARAM_TARGET_MENTION` | `"ToMentionType"` |

| edu.uchsc.ccp.uima.ae.util.converter.DocumentLevelAnnotationCreator\_AE | | |
| --- | --- | --- |
| `public static final String` | `PARAM_ANNOTATION_TYPE_TO_CONVERT` | `"AnnotationTypeToConvert"` |
| `public static final String` | `PARAM_ANNOTATORS_TO_IGNORE` | `"AnnotatorsToIgnore"` |
| `public static final String` | `PARAM_REQUIRED_SLOTS` | `"RequiredSlots"` |

| edu.uchsc.ccp.uima.ae.util.filter.AnnotationConsensusFilter\_AE | | |
| --- | --- | --- |
| `public final String` | `PARAM_ANNOTATION_SETS_TO_IGNORE` | `"AnnotationSetsToIgnore"` |
| `public final String` | `PARAM_ANNOTATION_TYPE_OF_INTEREST` | `"AnnotationTypeOfInterest"` |
| `public static final String` | `PARAM_CONSENSUS_THRESHOLD` | `"ConsensusThreshold"` |

| edu.uchsc.ccp.uima.ae.util.filter.AnnotationSetFilter\_AE | | |
| --- | --- | --- |
| `public static final String` | `PARAM_ANNOTATIONSET_IDS_TO_KEEP_LIST` | `"AnnotationSetIDsToKeepList"` |

| edu.uchsc.ccp.uima.ae.util.filter.AnnotatorFilter\_AE | | |
| --- | --- | --- |
| `public static final String` | `PARAM_ANNOTATOR_IDS_TO_KEEP_LIST` | `"AnnotatorIDsToKeepList"` |

| edu.uchsc.ccp.uima.ae.util.filter.ClassMentionRemovalFilter\_AE | | |
| --- | --- | --- |
| `public static final String` | `PARAM_CLASS_MENTION_TYPES_TO_REMOVE_LIST` | `"ClassMentionTypesToRemoveList"` |

| edu.uchsc.ccp.uima.ae.util.filter.ClassMentionTypeFilter\_AE | | |
| --- | --- | --- |
| `public static final String` | `PARAM_CLASS_MENTION_TYPES_TO_KEEP_LIST` | `"ClassMentionTypesToKeepList"` |

| edu.uchsc.ccp.uima.ae.util.filter.OverlappingAnnotationResolver\_AE | | |
| --- | --- | --- |
| `public final String` | `PARAM_ANNOTATION_SETS_TO_IGNORE` | `"AnnotationSetsToIgnore"` |
| `public final String` | `PARAM_ANNOTATION_TYPE_OF_INTEREST` | `"AnnotationTypeOfInterest"` |
| `public final String` | `PARAM_KEEP_LONGEST_COMMON_SUBSTRING` | `"KeepLongestCommonSubstring"` |

| edu.uchsc.ccp.uima.ae.util.filter.SlotRemovalFilter\_AE | | |
| --- | --- | --- |
| `public static final String` | `PARAM_SLOTS_TO_REMOVE_LIST` | `"SlotsToRemoveList"` |

| edu.uchsc.ccp.uima.cc.summary.AnnotationPrinter | | |
| --- | --- | --- |
| `public final String` | `PARAM_OUTPUT_INTO_MULTIPLE_FILES` | `"OutputInToMultipleFiles"` |
| `public final String` | `PARAM_OUTPUTFILENAME` | `"OutputFileName"` |
| `public final String` | `PARAM_OUTPUTTOFILE` | `"OutputToFile"` |
| `public final String` | `PARAM_USE_ALT_EVAL_FORMAT` | `"UseAltEvalFormat"` |
| `public final String` | `PARAM_USE_ANNOTATION_TO_FILE_FORMAT` | `"UseAnnotationToFileFormat"` |
| `public final String` | `PARAM_USE_BIOCREATIVEFORMAT` | `"UseBiocreativeFormat"` |
| `public final String` | `PARAM_USE_BIOCREATIVENORMFORMAT` | `"UseBiocreativeNormFormat"` |
| `public final String` | `PARAM_USE_BRILLFORMAT` | `"UseBrillFormat"` |
| `public final String` | `PARAM_USE_EMBEDDEDXMLFORMAT` | `"UseEmbeddedXMLFormat"` |
| `public final String` | `PARAM_USE_MUTATION_OUTPUT_FORMAT` | `"UseMutationOutputFormat"` |
| `public final String` | `PARAM_USE_ONELINE_FORMAT` | `"UseOneLineOutputFormat"` |
| `public final String` | `PARAM_USE_SIMPLE_COLLECTION_READER_FORMAT` | `"UseSimpleCollectionReaderFormat"` |
| `public final String` | `PARAM_USE_THING_WITH_ENTREZ_ID_FORMAT` | `"UseThingWithEntrezIDFormat"` |
| `public final String` | `PARAM_USE_WORDFREAKFORMAT` | `"UseWordFreakFormat"` |

| edu.uchsc.ccp.uima.cc.summary.comparison.AnnotationComparator\_CC | | |
| --- | --- | --- |
| `public static final String` | `PARAM_ANNOTATION_OUTPUT_FILE` | `"AnnotationOutputFile"` |
| `public static final String` | `PARAM_CONFIG_FILE` | `"ConfigFile"` |
| `public static final String` | `PARAM_USE_IDENTICAL_MENTION_COMPARATOR` | `"UseIdenticalMentionComparator"` |
| `public static final String` | `PARAM_USE_IGNORE_SPAN_COMPARATOR` | `"UseIgnoreSpanComparator"` |
| `public static final String` | `PARAM_USE_OVERLAP_SPAN_COMPARATOR` | `"UseOverlapSpanComparator"` |
| `public static final String` | `PARAM_USE_SHARED_END_SPAN_COMPARATOR` | `"UseSharedEndSpanComparator"` |
| `public static final String` | `PARAM_USE_SHARED_START_OR_END_SPAN_COMPARATOR` | `"UseSharedStartOrEndSpanComparator"` |
| `public static final String` | `PARAM_USE_SHARED_START_SPAN_COMPARATOR` | `"UseSharedStartSpanComparator"` |
| `public static final String` | `PARAM_USE_STRICT_SPAN_COMPARATOR` | `"UseStrictSpanComparator"` |

| edu.uchsc.ccp.uima.util.UIMA\_Annotation\_Util | | |
| --- | --- | --- |
| `public static final boolean` | `DEBUG` | `false` |

| edu.uchsc.ccp.uima.util.UIMASyntacticAnnotation\_Util | | |
| --- | --- | --- |
| `public static final boolean` | `DEBUG` | `false` |

| edu.uchsc.ccp.util.external.penn.PennBioTaggerEISystem | | |
| --- | --- | --- |
| `public static final String` | `TAGGER_TYPE_GENE` | `"gene"` |
| `public static final String` | `TAGGER_TYPE_MALIGNANCY` | `"malignancy"` |
| `public static final String` | `TAGGER_TYPE_VARIATION` | `"variation"` |

| edu.uchsc.ccp.util.nlp.document.DocumentSectionTypes | | |
| --- | --- | --- |
| `public static final int` | `ABSTRACT_ID` | `2` |
| `public static final String` | `ABSTRACT_NAME` | `"abstract"` |
| `public static final int` | `HW_ABSTRACT` | `20` |
| `public static final int` | `HW_TITLE` | `20` |
| `public static final int` | `OTHER_ID` | `0` |
| `public static final String` | `OTHER_NAME` | `"other"` |
| `public static final int` | `TITLE_ID` | `1` |
| `public static final String` | `TITLE_NAME` | `"title"` |

| edu.uchsc.ccp.util.nlp.mention.ClassMentionTypes | | |
| --- | --- | --- |
| `public static final String` | `ALANINE` | `"Alanine, Ala, A"` |
| `public static final String` | `AMINO_ACID` | `"amino acid"` |
| `public static final String` | `ARGININE` | `"Arginine, Arg, R"` |
| `public static final String` | `ASPARAGINE` | `"Asparagine, Asn, N"` |
| `public static final String` | `ASPARTATE` | `"Aspartate, Asp, D"` |
| `public static final String` | `BIOLOGICAL_SEQUENCE` | `"biological sequence"` |
| `public static final String` | `BIOLOGICAL_SEQUENCE_ELEMENT` | `"biological sequence element"` |
| `public static final String` | `BIOLOGICAL_SEQUENCE_POSITION` | `"biological sequence position"` |
| `public static final String` | `BIOLOGICAL_SUBSEQUENCE` | `"biological subsequence"` |
| `public static final String` | `CELL_LINE` | `"cell line"` |
| `public static final String` | `CELL_TYPE` | `"cell"` |
| `public static final String` | `CLASS_MENTION_TYPE` | `"class mention"` |
| `public static final String` | `CLAUSE` | `"clause"` |
| `public static final String` | `CYSTEINE` | `"Cysteine, Cys, C"` |
| `public static final String` | `DELETION` | `"deletion"` |
| `public static final String` | `DISEASE` | `"disease"` |
| `public static final String` | `DNA` | `"DNA"` |
| `public static final String` | `DOCUMENT_FIGURE_LEGEND` | `"figure caption"` |
| `public static final String` | `DOCUMENT_SECTION` | `"section"` |
| `public static final String` | `DOCUMENT_SECTION_HEADING` | `"section heading"` |
| `public static final String` | `DOCUMENT_SUBSECTION` | `"subsection"` |
| `public static final String` | `DOCUMENT_SUBSECTION_HEADING` | `"subsection heading"` |
| `public static final String` | `DOCUMENT_TABLE_LEGEND` | `"table caption"` |
| `public static final String` | `DOCUMENT_TITLE` | `"title"` |
| `public static final String` | `EXPRESSION` | `"expression"` |
| `public static final String` | `GENE` | `"gene"` |
| `public static final String` | `GENE_GENERIC` | `"generic gene"` |
| `public static final String` | `GENE_OR_GENE_PRODUCT` | `"gene or gene product"` |
| `public static final String` | `GENIA_atom` | `"atom"` |
| `public static final String` | `GENIA_body_part` | `"body part"` |
| `public static final String` | `GENIA_carbohydrate` | `"carbohydrate"` |
| `public static final String` | `GENIA_cell_component` | `"cell component"` |
| `public static final String` | `GENIA_DNA_domain_or_region` | `"DNA domain or region"` |
| `public static final String` | `GENIA_DNA_family_or_group` | `"DNA family or group"` |
| `public static final String` | `GENIA_DNA_NA` | `"GENIA DNA N/A"` |
| `public static final String` | `GENIA_DNA_substructure` | `"DNA substructure"` |
| `public static final String` | `GENIA_inorganic` | `"inorganic"` |
| `public static final String` | `GENIA_lipid` | `"lipid"` |
| `public static final String` | `GENIA_mono_cell` | `"mono cell"` |
| `public static final String` | `GENIA_multi_cell` | `"multi cell"` |
| `public static final String` | `GENIA_nucleotide` | `"nucleotide"` |
| `public static final String` | `GENIA_other_artificial_source` | `"GENIA other artificial source"` |
| `public static final String` | `GENIA_other_name` | `"GENIA other name"` |
| `public static final String` | `GENIA_other_organic_compound` | `"GENIA other organic compound"` |
| `public static final String` | `GENIA_peptide` | `"peptide"` |
| `public static final String` | `GENIA_polynucleotide` | `"polynucleotide"` |
| `public static final String` | `GENIA_protein_domain_or_region` | `"protein domain or region"` |
| `public static final String` | `GENIA_protein_family_or_group` | `"protein family or group"` |
| `public static final String` | `GENIA_protein_NA` | `"GENIA protein N/A"` |
| `public static final String` | `GENIA_protein_substructure` | `"protein substructure"` |
| `public static final String` | `GENIA_protein_subunit` | `"protein subunit"` |
| `public static final String` | `GENIA_RNA_domain_or_region` | `"RNA domain or region"` |
| `public static final String` | `GENIA_RNA_family_or_group` | `"RNA family or group"` |
| `public static final String` | `GENIA_RNA_NA` | `"GENIA RNA N/A"` |
| `public static final String` | `GENIA_RNA_substructure` | `"RNA substructure"` |
| `public static final String` | `GLUTAMATE` | `"Glutamate, Glu, E"` |
| `public static final String` | `GLUTAMINE` | `"Glutamine, Gln, Q"` |
| `public static final String` | `GLYCINE` | `"Glycine, Gly, G"` |
| `public static final String` | `HISTIDINE` | `"Histidine, His, H"` |
| `public static final String` | `HTML_PARAGRAPH` | `"html paragraph"` |
| `public static final String` | `HTML_PARAGRAPH_END` | `"paragraph end"` |
| `public static final String` | `HTML_PARAGRAPH_START` | `"paragraph start"` |
| `public static final String` | `HTML_PARAGRAPH_TAG` | `"paragraph tag"` |
| `public static final String` | `INSERTION` | `"insertion"` |
| `public static final String` | `ISOLEUCINE` | `"Isoleucine, Ile, I"` |
| `public static final String` | `LEUCINE` | `"Leucine, Leu, L"` |
| `public static final String` | `LYSINE` | `"Lysine, Lys, K"` |
| `public static final String` | `MACROMOLECULE` | `"macromolecule"` |
| `public static final String` | `MALIGNANCY` | `"malignancy"` |
| `public static final String` | `METHIONINE` | `"Methionine, Met, M"` |
| `public static final String` | `MUTATION_EVENT` | `"mutation event"` |
| `public static final String` | `NOMINALIZATION` | `"nominalization"` |
| `public static final String` | `NOMINALIZATION_ACTIVATION` | `"Activation"` |
| `public static final String` | `NOMINALIZATION_DEVELOPMENT` | `"Development"` |
| `public static final String` | `NOMINALIZATION_EXPRESSION` | `"Expression"` |
| `public static final String` | `NOMINALIZATION_FUNCTION` | `"Function"` |
| `public static final String` | `NOMINALIZATION_GROWTH` | `"Growth"` |
| `public static final String` | `NOMINALIZATION_INHIBITION` | `"Inhibition"` |
| `public static final String` | `NOMINALIZATION_INTERACTION` | `"Interaction"` |
| `public static final String` | `NOMINALIZATION_MUTATION` | `"Mutation"` |
| `public static final String` | `NOMINALIZATION_PHOSPHORYLATION` | `"Phosphorylation"` |
| `public static final String` | `NOMINALIZATION_REGULATION` | `"Regulation"` |
| `public static final String` | `NOMINALIZATION_RESPONSE` | `"Response"` |
| `public static final String` | `NOMINALIZATION_TRANSCRIPTION` | `"Transcription"` |
| `public static final String` | `NUCLEIC_ACID` | `"nucleic acid"` |
| `public static final String` | `ORGAN` | `"organ"` |
| `public static final String` | `ORGANISM` | `"organism"` |
| `public static final String` | `PDG_ACTION` | `"action"` |
| `public static final String` | `PHENYLALANINE` | `"Phenylalanine, Phe, F"` |
| `public static final String` | `PHRASE` | `"phrase"` |
| `public static final String` | `POLYPEPTIDE_SEQUENCE` | `"polypeptide sequence"` |
| `public static final String` | `POLYPEPTIDE_SUBSEQUENCE` | `"polypeptide subsequence"` |
| `public static final String` | `PROLINE` | `"Proline, Pro, P"` |
| `public static final String` | `PROTEIN` | `"protein"` |
| `public static final String` | `PROTEIN_ACRONYM` | `"acronym"` |
| `public static final String` | `PROTEIN_COMPLEX` | `"protein complex"` |
| `public static final String` | `PROTEIN_COMPOUND` | `"compound protein"` |
| `public static final String` | `PROTEIN_LONG_FORM` | `"long form protein"` |
| `public static final String` | `RNA` | `"RNA"` |
| `public static final String` | `SENTENCE` | `"sentence"` |
| `public static final String` | `SERINE` | `"Serine, Ser, S"` |
| `public static final String` | `SUBCELLULAR_LOCATION` | `"subcellular location"` |
| `public static final String` | `SUBSTITUTION` | `"substitution"` |
| `public static final String` | `TAXON` | `"taxon"` |
| `public static final String` | `THREONINE` | `"Threonine, Thr, T"` |
| `public static final String` | `TISSUE` | `"tissue"` |
| `public static final String` | `TOKEN` | `"token"` |
| `public static final String` | `TRANSPORT_GATED` | `"gated transport"` |
| `public static final String` | `TRYPTOPHAN` | `"Tryptophan, Trp, W"` |
| `public static final String` | `TYROSINE` | `"Tyrosine, Tyr, Y"` |
| `public static final String` | `UNDEFINED` | `"undefined"` |
| `public static final String` | `VALINE` | `"Valine, Val, V"` |
| `public static final String` | `VARIATION_EVENT` | `"variation-event"` |
| `public static final String` | `VARIATION_TYPE` | `"variation-type"` |
| `public static final String` | `VIRUS` | `"virus"` |

| edu.uchsc.ccp.util.nlp.mention.ComplexSlotMentionTypes | | |
| --- | --- | --- |
| `public static final String` | `BIOLOGICAL_SEQUENCE_ELEMENT_POSITION_IN_SEQUENCE` | `"position in sequence"` |
| `public static final String` | `BIOLOGICAL_SUBSEQUENCE_END_ELEMENT` | `"end element"` |
| `public static final String` | `BIOLOGICAL_SUBSEQUENCE_START_ELEMENT` | `"start element"` |
| `public static final String` | `COMPLEX_SLOT_MENTION_TYPE` | `"complex slot mention"` |
| `public static final String` | `DELETION_DELETED_ELEMENT` | `"deleted element"` |
| `public static final String` | `INSERTION_INSERTED_ELEMENT` | `"inserted element"` |
| `public static final String` | `INSERTION_INSERTION_START` | `"insertion start"` |
| `public static final String` | `SUBSTITUTION_MUTANT_ELEMENT` | `"mutant element"` |
| `public static final String` | `SUBSTITUTION_WILDTYPE_ELEMENT` | `"wild type element"` |

| edu.uchsc.ccp.util.nlp.mention.SlotMentionTypes | | |
| --- | --- | --- |
| `public static final String` | `BIOLOGICAL_SEQUENCE_SEQUENCE` | `"sequence"` |
| `public static final String` | `CLAUSE_LEVEL` | `"clauseLevel"` |
| `public static final String` | `CLAUSE_NUMBER` | `"clauseNumber"` |
| `public static final String` | `CLAUSE_TYPE` | `"clauseType"` |
| `public static final String` | `DOCUMENT_SECTION_NAME` | `"section name"` |
| `public static final String` | `DOCUMENT_SUBSECTION_NAME` | `"subsection name"` |
| `public static final String` | `EXPRESSION_EXPRESSED_GENE_OR_GENE_PRODUCT` | `"expressed gene or gene product"` |
| `public static final String` | `EXPRESSION_LOCATION` | `"location"` |
| `public static final String` | `FIGURE_ID` | `"figure id"` |
| `public static final String` | `MEDLEE_ID` | `"MedLEE ID"` |
| `public static final String` | `MESH_ID` | `"MeshID"` |
| `public static final String` | `NCBI_TAX_ID` | `"NCBI Taxon ID"` |
| `public static final String` | `PART_OF` | `"part of"` |
| `public static final String` | `PHRASE_LEVEL` | `"phraseLevel"` |
| `public static final String` | `PHRASE_NUMBER` | `"phraseNumber"` |
| `public static final String` | `PHRASE_TYPE` | `"phraseType"` |
| `public static final String` | `PROCESSED_TEXT_SLOT` | `"processed text"` |
| `public static final String` | `PROTEIN_ENTREZGENEID` | `"entrez_gene_id"` |
| `public static final String` | `SLOT_MENTION_TYPE` | `"slot mention"` |
| `public static final String` | `TABLE_ID` | `"table id"` |
| `public static final String` | `TOKEN_IS_ANAPHOR_FOR` | `"isAnaphorFor"` |
| `public static final String` | `TOKEN_LEMMA` | `"lemma"` |
| `public static final String` | `TOKEN_NUMBER` | `"tokenNumber"` |
| `public static final String` | `TOKEN_PARTOFSPEECH` | `"partOfSpeech"` |
| `public static final String` | `TOKEN_STEM` | `"stem"` |
| `public static final String` | `TOKEN_TYPED_DEPENDENCY` | `"typedDependency"` |

| edu.uchsc.ccp.util.nlp.mention.comparison.MentionComparator | | |
| --- | --- | --- |
| `protected final int` | `MULTIPLIER` | `3` |

| edu.uchsc.ccp.util.nlp.parser.corpus.Bio1CorpusParser | | |
| --- | --- | --- |
| `public static final int` | `DOCUMENT_COLLECTION_ID` | `8` |

| edu.uchsc.ccp.util.nlp.parser.corpus.BioIECorpusParser | | |
| --- | --- | --- |
| `public static final int` | `DOCUMENT_COLLECTION_ID` | `11` |

| edu.uchsc.ccp.util.nlp.parser.corpus.GeniaCorpusParser | | |
| --- | --- | --- |
| `public static final int` | `DOCUMENT_COLLECTION_ID` | `4` |

| edu.uchsc.ccp.util.nlp.parser.corpus.IProLinkCorpus1Parser | | |
| --- | --- | --- |
| `public static final int` | `DOCUMENT_COLLECTION_ID` | `9` |

| edu.uchsc.ccp.util.nlp.parser.corpus.IProLinkCorpus2Parser | | |
| --- | --- | --- |
| `public static final int` | `DOCUMENT_COLLECTION_ID` | `9` |

| edu.uchsc.ccp.util.nlp.parser.corpus.TexasCorpusParser | | |
| --- | --- | --- |
| `public static final int` | `DOCUMENT_COLLECTION_ID` | `12` |

| edu.uchsc.ccp.util.nlp.parser.corpus.YapexCorpusParser | | |
| --- | --- | --- |
| `public static final int` | `DOCUMENT_COLLECTION_ID` | `7` |

---


|  |  |  |
| --- | --- | --- |
| **Overview** \| Package \| Class \| **Tree** \| **Deprecated** \| **Index** \| **Help** | |  |
| PREV   NEXT | **FRAMES**    **NO FRAMES**     **All Classes** |  |


---
